# Analgesic Mechanism of Sinomenine against Chronic Pain

**DOI:** 10.1155/2020/1876862

**Published:** 2020-05-06

**Authors:** Wei Jiang, Weiming Fan, Tianle Gao, Tao Li, Zhenming Yin, Huihui Guo, Lulu Wang, Yanxing Han, Jian-Dong Jiang

**Affiliations:** ^1^Zhejiang Zhenyuan Pharmaceutical Co., Ltd, 1015 West Shengli Road, Shaoxing, Zhejiang 312000, China; ^2^State Key Laboratory of Bioactive Substances and Function of Natural Medicine, Institute of Materia Medica, Chinese Academy of Medical Sciences, Beijing 100050, China; ^3^Beijing Key Laboratory of Traditional Chinese Medicine Basic Research on Prevention and Treatment of Major Diseases, Experimental Research Center, China Academy of Chinese Medical Sciences, Beijing 100700, China

## Abstract

Purified from the roots of the plant *Sinomenium acutum*, sinomenine is traditionally used in China and Japan for treating rheumatism and arthritis. Previously, we have demonstrated that sinomenine possessed a broad analgesic spectrum in various chronic pain animal models and repeated administration of sinomenine did not generate tolerance. In this review article, we discussed sinomenine's analgesic mechanism with focus on its role on immune regulation and neuroimmune interaction. Sinomenine has distinct immunoregulative properties, in which glutamate, adenosine triphosphate, nitric oxide, and proinflammatory cytokines are thought to be involved. Sinomenine may alter the unbalanced neuroimmune interaction and inhibit neuroinflammation, oxidative stress, and central sensitization in chronic pain states. In conclusion, sinomenine has promising potential for chronic pain management in different clinical settings.

## 1. Introduction

**Figure 1:**
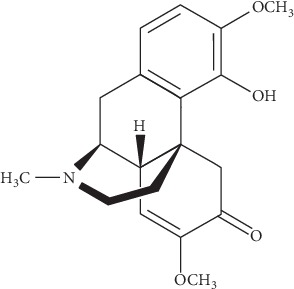
Chemical structure of sinomenine.

Traditional Chinese medicine (TCM) has a long history with rich clinical experience in pain management, and it also harbors a rich source of potential drug candidates. In particular, compared to Western medicines, TCM such as sinomenine (Figure 1) usually possesses similar efficacy but with fewer side effects. Purified from the roots of the climbing plant *Sinomenium Acutum*, sinomenine was originally found to be effective in treating rheumatism in Japan since the early 1930s [1]. It can also work as an immune suppressor since it inhibits lymphocyte proliferation and the synthesis of B-cell antibodies or immunoglobulin *G* in cells or animals [2, 3]. In the late 1990s, sinomenine hydrochloride (molecular weight, 365 Da; logP = 1.34; pKa = 7.98; saturated solubility in water, 118 mg/mL; saturated solubility in ethanol,11.4 mg/mL) has been developed as a widely used drug in China (Zhengqing Fengtongning sustained-release tablets) for rheumatoid arthritis therapy.

In addition to the aspect of antirheumatism, recent studies also demonstrated sinomenine's efficacy in alleviating pain. Compared with nonsteroidal anti-inflammatory drugs (NSAIDs), sinomenine was more efficacious in amelioration of morning stiffness and painful joints in patients with rheumatoid arthritis [4]. Moreover, sinomenine (administered orally or subcutaneously) was remarkably beneficial in alleviating pain in many types of neuralgia, such as sciatic neuritis and lumbalgia [1]. In previous study, we have demonstrated that sinomenine possessed promising analgesic properties in various experimental chronic pain models [5–8]. Furthermore, repeated administration of sinomenine did not generate tolerance but increased the baseline pain threshold [6], and pretreatment of sinomenine could delay the morphine tolerance [9], indicating its potential when applied in a long term. Besides, sinomenine also exhibited anxiolytic-like effect that resembles the partial 5-HT_1A_ agonist gepirone [10].

In terms of acute toxicity, sinomenine elicits convulsant-type central excitation at large doses, which can be also seen in morphine and its surrogates. However, sinomenine is less dangerous compared with opioids, due to the absence of central inhibitory effects, albeit high dose (160 mg/kg, intraperitoneal) of sinomenine could generate sedation and decrease motor activity [1]. In rats, LD_50_ of sinomenine is 535 ± 4 mg/kg or 580 ± 51 mg/kg for intraperitoneal or subcutaneous application, respectively [1]. When sinomenine was applied in the long term (at 150 mg/kg/day for 6 consecutive weeks), no irreversible organic damage could be generated [11].

Sinomenine has distinct immunoregulative properties (Table 1) in which glutamate, nitric oxide (NO), proinflammatory cytokines, and markers of oxidative stress are thought to be involved. As the paradigm chronic pain treatment is switching from single target towards an entire network, in the following paragraph, we discussed sinomenine's analgesic mechanism with focus on its potential roles in immune regulation and neuroimmune interaction.

## 2. Altered Neurotransmission by Sinomenine

Excessive extracellular levels of glutamate trigger the N-methyl-D-aspartate (NMDA) receptor facilitated neuronal toxicity and the maintenance of chronic pain [36]. Sinomenine can effectively reduce outrageous glutamate levels in the striatum of rats with spared nerve injury and alleviate the pain behaviors in the same animals at the same time [12]. In addition, sinomenine decreases the elevated levels of cyclic adenosine monophosphate (cAMP), noradrenaline, and intracellular Ca^2+^, while restoring serotonin and dopamine levels to normal states in morphine-dependent, naloxone-precipitated withdrawal rats [13]. Such an outcome was achieved by inhibition of P-NMDAR1/NMDAR1 expression via downregulating calmodulin-dependent protein kinase II (CAMKII) and phosphorylated cAMP-response element-binding protein (P-CREB) pathways [35]. These examples indicate that sinomenine may mitigate the neurotoxicity resulted from chronic pain or morphine tolerance.

There is evidence suggests sinomenine is an opioid *μ*-receptor modulator, based on it can dose-dependently replace the binding of [^3^H] naloxone in Chinese hamster ovary cells transfected with the opioid *μ*-receptor [9]. Sinomenine's modulatory effect on opioid *μ*-receptors may be relevant to its ability to prevent the development of morphine tolerance [9]. However, in our experience, sinomenine's analgesic effect is independent of opioid receptors [5, 8], since naloxone cannot prevent or reverse the antiallodynic effect of sinomenine. However, other mechanisms, such as activating the gamma-aminobutyric acid A (GABA_A_) receptor [37] or blocking the acid-sensing ion channel/calcium channels [38], could account for sinomenine's inhibitory effect on neuronal overactivation.

## 3. Role of Histamine on Sinomenine's Antihyperalgesic Effect

Histamine has been known to act on sensory neurons to produce itch, and histamine-induced itch converts to pain in states of neuropathic hyperalgesia [39]. In rodents, histamine seems to have both pro and antinociceptive properties. For instance, daily subcutaneous injection of histamine in mice can generate analgesia which reached maximum after 6–8 days [1]. However, high dose of intrathecally injected histamine (1600pmol) can evoke nociceptive behaviors consisting of biting/licking along with occasional scratching in mice [19]. Histamine receptors have differed roles in chronic pain. Specifically, following nerve injury, blocking H1 or H2 receptors but activating the H4 receptor generated therapeutic effect, while an H3 and H4 receptor blocker facilitated hypersensitivity [40]. Taking selective H3 antagonists is effective in reversing neuropathic pain [41] also into consideration, we can generally conclude that activating H1, H2, and H3 receptors are pronociceptive, but activation of the H4 receptor is antinociceptive in chronic pain states.

Sinomenine has a potent histamine-releasing ability associated with degranulation of mast cells in mammalian tissues [1]. In order to test if sinomenine's antinociceptive effects against chronic pain is mediated through secondary histamine release, we blocked histamine receptors during sinomenine application in neuropathic pain models [5]. However, analgesic efficacy of sinomenine remained unaltered under such blockage of histamine pathways. Thus, histamine is irrelative to sinomenine's antihyperalgesic efficacy.

## 4. Sinomenine Could Be a NO and Selective iNOS Inhibitor

Nitric oxide (NO) is a messenger molecule synthesized by three isoforms of nitric oxide synthases (NOSs), which diffuses from the site of production across cellular membranes. All three isoforms of NOS are present in the nervous system, namely, the constitutive neuronal (nNOS), endothelial (eNOS), and the inducible (iNOS) isoforms. Under normal conditions, iNOS is mainly absent in neural tissues but is upregulated during inflammation, while eNOS is present in the brain vasculature and nNOS is present in the spinal cord [42].

There is an essential role played by the NO system in the development of chronic pain. Clinically, a significant increase in the plasma levels of nitrate/nitrite concentrations (used as an indicator of NO synthesis) has been noted in chronic pain patients in comparison with healthy individuals. In animal studies, upregulation of nNOS at messenger RNA or protein levels has been detected in large neurons of laminae III and IV after sciatic nerve transection (SNT), while the selective inhibitor of nNOS blocked hyperalgesia and allodynia following SNT [42]. NO can also facilitate neuropathic pain by enhancing cyclooxygenase (COX) activity to promote the synthesis of prostaglandin E2 (PGE2), which can be reversed by application of the nonselective COX inhibitor [43]. In addition, NO activates NMDA receptors to promote the development and maintenance of central sensitization, while spinally administered NOS inhibitors suppressed formalin- or carrageenan-induced hypersensitivities [42].

Notably, beneficial effects of nonselective NOS inhibitors were limited, as they also suppress eNOS expression and therefore antagonize its protective effects. In certain circumstances, the nonselective NOS inhibitor even aggravates ischemic pain [42]. Sinomenine can potently reduce the synthesis of NO and selectively inhibit iNOS and nNOS expression in activated macrophages/microglia cells and the cerebral cortex, respectively [13, 14, 21, 22]. These properties of sinomenine give it an advantage over nonselective NOS inhibitors, since selective suppression of iNOS and nNOS is a better strategy for controlling chronic pain [44].

## 5. Inhibition of COX2/PGE2 by Sinomenine

Once a nerve is damaged, Wallerian degeneration occurs that subsequently results in activation of Schwann cells and an influx of inflammatory cells into injured nerve tissue. These infiltrated cells produce a wide array of inflammatory mediators. Cyclooxygenase 2-dependant prostaglandin E2 (COX2/PGE2) is one of the most important mediators, which abundantly produced in the nerve injury site and involved in the genesis of neuropathic pain. COX2/PGE2 can promote neuropathic pain by upregulating the iNOS in macrophages, which could be reversed by nonselective COX inhibitors [43]. Besides, PGE2 overproduced nerves contribute to the maintenance of neuropathic pain by chronic activation on nociceptors and enhancing the synthesis of pain-facilitatory mediators in DRG neurons. Preclinical and clinical studies demonstrated that COX2 is dramatically upregulated in invading macrophages and Schwann cells in injured nerves following various types of nerve injury, and such upregulation can even last for years [43].

Sinomenine can potently suppress the pronociceptive PGE2 synthesis through COX2 inhibition in lipopolysaccharide-treated macrophages [21] and in enriched microglia cells [22]. It is reasonable that sinomenine may attenuate neuropathic pain by downregulating the activity of COX2/PGE2.

## 6. Reduction of TNF by Sinomenine

The tumor necrosis factor (TNF) is an inflammatory cytokine that involved in the development of neuropathic pain. Using the standard model of chronic constriction injury (CCI) of the sciatic nerve in rats, TNF has been detected at the injury site and shows temporal upregulation [45]. Interestingly, such elevation of TNF is bilateral, not only in the ipsilateral DRG but also in the contralateral DRG unassociated with the injured nerve (Jancalek et al., 2010). Moreover, intrasciatic injection of TNF in rat produces hypersensitivity, which is similar to neuropathic pain in humans [45]. It has been shown that TNF can enhance the activity of tetrodotoxin-resistant sodium channels in nociceptive DRG neurons as well as increase membrane K^+^ ion conductance in a non-voltage-gated fashion leading to overall neuronal hyperexcitability and hence facilitates neuropathic pain [45].

Besides, multiple studies have demonstrated that clinically administration of agents that antagonize TNF-attenuated chronic pain [46, 47]. Other studies also suggested that inhibition of TNF prevents the development of neuropathic pain through dampening of spinal p38 MAPK activation [48]. Notedly, TNF also interacts with the norepinephrine system in the central nervous system. Norepinephrine has an antiallodynic effect through the activation of the adrenaline *α*2 receptor. During chronic pain, inhibition of norepinephrine release in the hippocampus by TNF is significantly enhanced, which could be reversed by the TNF blockade. Interestingly, the norepinephrine inhibitory function of TNF transforms to facilitation following chronic administration of antidepressant drugs or during the natural dissipation of hyperalgesia [49].

A clinical study also indicated that nerve biopsies of human patients with painful neuropathy show higher levels of TNF expression, especially in Schwann Cells [50]. In addition, TNF used as a clinical anticancer treatment led to peripheral neuropathy [45]. Since sinomenine can effectively reduce the TNF levels in activated microglia and microphages [21, 23], it is possible that sinomenine's analgesic mechanism is partially mediated through downregulating the activity of TNF, albeit merely deplete TNF alone cannot abolish neuropathic pain, as has already been demonstrated by the failure of the TNF antibody in clinical trials for sciatica [45]. However, it is also of interests to know if sinomenine has the ability to affect the norepinephrine system by manipulating TNF levels.

## 7. Sinomenine Inhibits INF-*γ*, IL-6, IL-1*β*, and IL-4

Interferon gamma (IFN-*γ*) is an inflammatory cytokine that associated with a number of autoimmune diseases. The immunemodulatory role of INF-*γ* involves activation of macrophages to produce NO/TNF and upregulates the major histocompatibility complex (MHC) antigens [51]. It has been known that intrathecally injected INF-*γ* can facilitate the nociceptive flexor reflex in rats [52], and locally administered INF-*γ* can induce thermal hyperalgesia [51]. Emerging new studies has enriched our knowledge about how INF-*γ* has participated in the establishment of chronic pain. Spinal microglia cells express the receptor for INF-*γ* (INF-*γ*R), which converts microglia into activated cells when being stimulated and produces a long-lasting pain hypersensitivity [52]. Ablating INF-*γ*R severely impairs periphery nerve injury-evoked microglia activation and tactile allodynia without affecting microglia in the contralateral dorsal horn or basal pain sensitivity [53]. Sinomenine was able to decrease serum levels of INF-*γ* in patients with mesangial proliferative nephritis [18]. It also suppressed the INF-*γ* and antibody production in spleen cells of CIA rats [32]. These evidences suggest that sinomenine may exert its antihyperalgesic effect by reducing the INF-*γ* level.

In response to never injury, microglia transforms into macrophage-like cells that express MHC antigens to secrete proinflammatory cytokines including IL-1*β* and IL-6. IL-1*β* and IL-6 are proinflammatory cytokines that can boost immune response and exacerbate symptoms of rheumatoid arthritis. Recent animal studies revealed the facilitatory role of IL-6 and IL-1*β* in the development of neuropathic pain. After chronic constriction injury (CCI) in the infraorbital nerve of rats, levels of IL-6 and IL-1*β* in the ventromedial medulla (RVM) were increased [45]. Injection of IL-6 and IL-1*β* into RVM increased NR1 phosphorylation of the NMDA receptor and subsequently generated hyperalgesia, which could be reversed by an NMDA antagonist [45]. In addition, injection of IL-6 induced microglial activation and resulted in mechanical allodynia and thermal hyperalgesia to a similar extent as the CCI model [54]. Furthermore, a clinical study also demonstrated that spinal cord injured patients exhibited higher serum concentrations of IL-6 and IL1-*β* than healthy subjects [55]. Sinomenine can suppress the production of IL1-*β* and IL-6 in macrophages and decrease the serum concentrations of IL1-*β* and IL-6 in CIA rats [26]. It is possible that sinomenine can ameliorate chronic inflammatory or neuropathic pain by reducing levels of IL-6 and IL1-*β*, thereby suppressing the activation of microglia cells.

IL-4 is a peptide that has both proinflammatory and antiallodynic properties. Inoculation of vector S4IL4 to express IL-4 in DRG neurons 1 week before sciatic nerve ligation (SNL) delayed the onset of thermal hyperalgesia and tactile allodynia in rats but did not prevent the ultimate development of chronic pain manifestations [56]. Treatment with sinomenine decreased production of IL-4 in antigen activated RBL-2H3 cells [23]. Thus, sinomenine was able to suppress the upregulation of IL-4 during inflammatory responses. However, the role of IL-4 in the analgesic effect induced by sinomenine remained largely uncertain.

## 8. Sinomenine Suppresses the Activity of P38 MAPK, MMPs, and NF-*κ*B

P38 mitogen-activated protein kinases (p38-MAPKs) are the mammalian orthologue of the yeast high-osmolarity glycerol response kinase which participates in a signaling cascade controlling cellular responses to cytokines and stress. Stimulation of microglia by proinflammatory cytokines and spinal astrocytes activation is mediate via the p38-MAPK system and directly contributed to central sensitization [45]. For instance, IL-6 induces microglial CX3C chemokine receptor 1 (CX3CR1) expression in the spinal cord after peripheral nerve injury through activating p38 MAPK, and treatment with the p38 MAPK-specific inhibitor suppressed CX3CR1 expression induced by CCI [54]. In addition, it has been found that spinal nerve ligation in rats leads to mechanical allodynia with concomitant rises in TNF and p38 MAPK phosphorylation. Such mechanical allodynia can be reduced after treatment with inhibitors of p38 MAPK. Similarly, in naïve rats, inhibition of spinal p38 MAPK activation abolishes hyperalgesia in response to the central infusion of IL-1*β* or substance P and prevents the development of neuropathic pain [48, 57, 58].

Following treatment with sinomenine, a significant decrease of the p38 MAPK activity has been seen in activated RBL-2H3 cells [23]. After neuronal damage, sinomenine can modulate microglia and macrophages in the nerve injury sites to inhibit p38 MAPK phosphorylation. Considering sinomenine exerts anti-inflammatory and neuroprotective effects through inhibition of microglial activation [22], it is expected that sinomenine may also promote the stabilization of the intracellular microenvironment and suppress neuronal overactivation in chronic pain situation [59].

Matrix metalloproteinases (MMPs) are zinc-dependent endopeptidases which degrade various kinds of extracellular matrix proteins. They are known to be involved in the synthesis of apoptotic ligands, chemokines, and cytokines [43]. Recent evidences suggest that MMPs have contributed to the development and maintenance of neuropathic pain. Following nerve injury, MMP-2 and MMP-9 are upregulated in Schwann cells, invading macrophages and injured axons. As verifying evidence, synthetic MMP inhibitors relieved many types of chronic pain [43]. Sinomenine can remarkably inhibit the elevated protein expressions and activities of MMP-2 and MMP-9 during inflammation [26], suggesting that sinomenine may also suppress the MMPs in peripheral or central nervous system that ultimately alleviates chronic pain.

The transcription factor nuclear factor kappa B (NF-*κ*B) is a key regulator of inflammatory responses. Inhibiting NF-*κ*B activity is beneficial in controlling both pain and inflammation. For instance, transgenic inhibition of NF-*κ*B in glial fibrillary acidic protein (GFAP) expressing glial cells attenuated pain and neuronal inflammation after peripheral nerve injury [60]. Sinomenine inhibits the maturation of monocyte-derived dendritic cells through inactivation of NF-*κ*B [24]. In addition, studies have shown that sinomenine can decrease the mRNA levels of TNF and IL-1*β* by inhibiting the NF-*κ*B binding activity, through upregulation of the inhibitor of kappa B (I*κ*B-*α*) expression in peritoneal macrophages and synoviocytes [24]. Thus, sinomenine may deactivate NF-*κ*B activity in nerve injury sites and improve neuropathic pain conditions.

## 9. Sinomenine May Decrease ROS Generation and Inhibit Central Sensitization

Reactive oxygen species (ROS) are chemically-reactive molecules containing oxygen. ROS are generated as a natural byproduct of the normal metabolism of oxygen and have important roles in cell signaling. However, during environmental stress (e.g., UV or heat exposure), ROS levels can increase dramatically and result in significant damage to cell structures. This phenomenon is known as oxidative stress.

Recent experimental data suggest that ROS are involved in the maintenance of chronic pain. In rats with neuropathic pain, the mechanical allodynia developed after nerve ligation was significantly reversed after treatment with ROS scavengers. In addition, the superoxide dismutase (SOD) mimetic which converts free-radical superoxide to hydrogen peroxide was very effective in reducing inflammation and hyperalgesia after carrageenan injection into the rat paw [61]. Moreover, mitochondrial ROS production is elevated in the spinal cord following nerve damage, while injection of ROS scavengers could effectively bring down the abnormal ROS levels and attenuate allodynia [62].

Central sensitization is defined by increased responsiveness of dorsal horn neurons to nociceptive peripheral stimulation [36]. Behavioral and electrophysiological studies indicate that ROS are involved in central sensitization. In animals with neuropathic pain, P-NMDR1 expression in the spinal cord was increased, and injection of the ROS scavenger dramatically blocked the enhancement of spinal P-NMDR1 and suppressed central sensitization [61]. Thus, ROS promote central sensitization via NMDA-receptor activation (which can be reversed by ROS scavengers).

Sinomenine exhibited anti-inflammatory properties by reducing superoxide ions, inhibiting the microglial nicotinamide adenine dinucleotide phosphate (NADPH) oxidase [22] and upregulated nuclear factor erythroid 2-related factor 2 (Nrf2). These effects resulted in the reduction of extracellular ATP and the intracellular ROS levels further prevented the NF-*κ*B activation and proinflammatory cytokine production [22].

Protein kinase B (PKB) is a serine protein kinase which can be stimulated by ROS to protect cells against oxidative stress. Studies have shown that PKB is activated in response to neuropathic pain, which subsequently acts on sensory neurons to stimulate the expression of nociception-related genes [62]. Sinomenine can suppress PKB activity in antigen-activated RBL-2H3 cells [23]. The above findings indicate the possibility that sinomenine may deactivate NMDA receptors and suppress central sensitization by decreasing ATP and ROS production in the central nervous system and reduce the expression of nociception-related genes via inhibition of PKB activity.

## 10. Neuroimmune Changes Induced by Sinomenine

**Figure 2:**
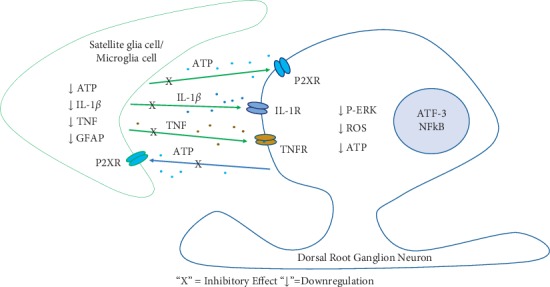
Sinomenine-induced peripheral neuroimmune interaction occurred at the dorsal root ganglion neurons. ATP, adenosine triphosphate; IL1-ß, interluekin1 ß; TNF, tumor necrosis factor; GFAP, glial fibrillary acidic protein; P2XR, P2X receptor; IL-1R, interluekin1 receptor; TNFR, tumor necrosis factor; P-ERK, phospho-extracellular regulated protein kinases; ROS, reactive oxygen species; ATF-3, activating transcription factor-3; NFkB, transcription factor nuclear factor kappa B.

**Figure 3:**
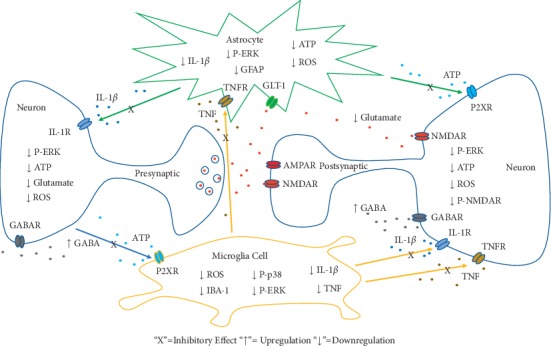
Sinomenine-induced central neuroimmune interaction occurred at the spinal dorsal horn. ATP, adenosine triphosphate; IL1-ß, interleukin1 ß; TNF, tumor necrosis factor; GFAP, glial fibrillary acidic protein; P2XR, P2X receptor; IL-1R, interleukin1 receptor; TNFR, tumor necrosis factor; P-ERK, phospho-extracellular regulated protein kinases; ROS, reactive oxygen species; ATF-3, activating transcription factor-3; GABA, *γ*-amino butyric acid; GABAR, *γ*-amino butyric acid receptor; P-p38, phospho-p38 mitogen-activated protein kinase; NMDAR, N-methyl-D-aspartate receptor; P-NMDAR, phosphor-N-methyl-D-aspartate receptor, IBA-1, ionized calcium-binding adapter molecule 1; GLT-1, glial glutamate transporter 1.

Pain signal is generated in the peripheral nerve terminals, through the afferent sensory nerve fibers, transmitted into the DRG (where the neurons of the peripheral nerves are located), and then transduced by the nerve fibers to the dorsal horn of the spinal cord. In the dorsal horn of the spinal cord, pain signals usually require pain-relay neurons to enter the higher levels of the nervous system (brainstem, midbrain, and cortex) to complete the process of pain perception [63]. Here, we proposed mechanisms for sinomenine induced neuroimmune changes in the DRG (Figure 2) and spinal dorsal horn (Figure 3).

In DRG (as shown in Figure 2), under sustained pain conditions, pain signals cause excessive adenosine triphosphate (ATP) production in DRG neurons, which later on activates surrounding satellite glial cells through ATP-gated P2X receptors, and result in extracellular efflux of inflammatory mediators such as TNF and IL-1ß [59]. These signaling molecules then act on neurons through their respective receptors, causing oxidative stress, as well as phosphorylation of phospho-extracellular regulated protein kinases (P-ERKs). The P-ERK subsequently enters the nucleus and causes activation of the ATF-3 transcription factor (a neuronal injury marker). Sinomenine may reduce the activated states of both DRG neurons and satellite glial cells by inhibiting the excessive ATP production and the release of inflammatory cytokines, which in turn prevent the activation of P-ERK and ATF-3.

In the spinal dorsal horn (as shown in Figure 2), under chronic pain situations, neurons are activated to produce excessive levels of ATP (and glutamate). Neuron-derived ATP drives the release of inflammatory mediators such as TNF and IL-1ß by activating P2X receptors in microglia cells. These inflammatory mediators then act back on neurons via their respective receptors, causing oxidative stress and phosphorylation of ERK. Alternatively, the inflammatory mediators can act on astrocytes to reduce the glutamate recycling, while releasing an excessive amount of ATP to promote their activated state (expressing P-ERK and GFAP). ATP released by astrocytes acts on neurons through P2X receptors and accelerates neuronal excitation through phosphorylation of NMDA receptors [36]. Excessive glutamate levels released by neurons then act on NMDA and *a*-amino-3-hydroxy-5-methylisoxazole-4-propionic acid (AMPA) receptors on the postsynaptic membrane, generating sustained activation in pain-relaying neurons, as well as producing cytotoxicity at the same time. In the dorsal horn, sinomenine can reduce the excessive ATP and glutamate release while enhancing GABAergic neurotransmission. These effects will subsequently help to suppress the production of inflammatory cytokines from microglial cells and astrocytes and decrease their active states (inhibiting P-ERK, P-p38, IBA-1, or GFAP expression). In turn, the dorsal horn neurons will be protected from harmful levels of glutamate and proinflammatory cytokines, and the ongoing sensitization in the same neurons will be suppressed.

## 11. Conclusion

**Figure 4:**
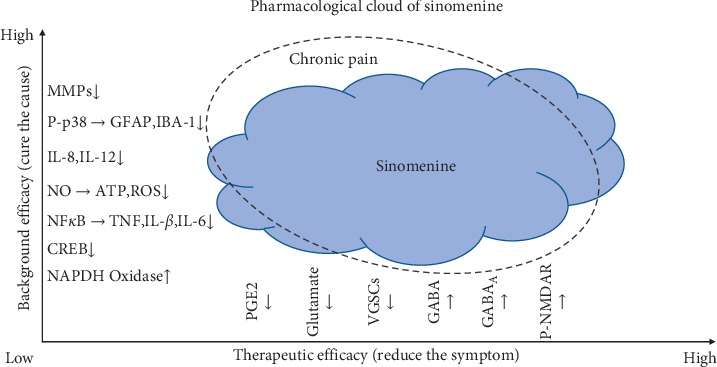
The pharmacological cloud of sinomenine. “↑” represents upregulation/activation; “↓” represents downregulation/inhibition. MMPs, matrix metalloproteinases; pP38, phospho-p38 mitogen-activated protein kinase; GFAP, glial fibrillary acidic protein; IBA-1, ionized calcium-binding adapter molecule 1; IL-8, interleukin 8; IL-12, interleukin 12; NO, nitric oxide; ATP, adenosine triphosphate; ROS, reactive oxygen species; NFkB, transcription factor nuclear factor kappa B; TNF, tumor necrosis factor; IL1-ß, interleukin1 ß; IL-6, interleukin 6; CREB, cAMP-response element-binding protein; PGE2, prostaglandin E2; VGSCs, voltage-gated sodium channels; P-NMDAR, phosphor-N-methyl-D-aspartate receptor.

To summarize, sinomenine is a rare anti-inflammatory and antihyperalgesic substance that can act on both peripheral and central nervous systems. Although it possessed different types pharmacological properties, no clear drug target has been found. So, sinomenine is likely to be a typical multitarget active substance (purified from TCM). To further describe the state of the art on the pro's and con's of potential sinomenine use in chronic pain therapy, we introduced a notion of “pharmacological cloud” (as shown in Figure 4). The clinical advantages of sinomenine (stronger analgesic efficacy with fewer side effects) are theoretically the result of its “pharmacological cloud” manifested in two dimensions: treating the symptoms of chronic pain (reducing physiological pain indicators) and treating the cause of chronic pain (reducing inflammation and changing neuroplasticity). In detail, sinomenine can block peripheral sodium channels, reduce glutamate levels, and activate GABA_A_ receptors, to exert a direct therapeutic effect stopping the firing of neurons; it can also alleviate the neuroinflammation and oxidative stress to reduce the activation of glial cells, thereby improving the external environment of neurons and achieving its background efficacy (altering unbalanced neuroimmune crosstalk, which is the cause of chronic pain). Although the application criteria of sinomenine need to be further verified before its widely usage, we conclude that sinomenine has promising potential for chronic pain management in different clinical settings.

## Figures and Tables

**Table 1:** The modulatory properties of sinomenine on neuroimmune regulators.

Substance	Type	Effect by sinomenine	Model/site of expression	Reference
Glutamate	Molecule	Decrease concentration	Brain extracellular fluid	[12]
GABA	Molecule	Decrease concentration	Brain extracellular fluid	[12]
Serotonin	Molecule	Increase concentration	Brain extracellular fluid	[13, 14]
Dopamine	Molecule	Increase concentration	Brain extracellular fluid	[13, 14]
cAMP	Molecule	Decrease concentration	Brain extracellular fluid	[13]
Opioid *μ* receptor	Protein	Dose-dependent activation	Chinese hamster ovary cell	[9]
Adenosine A_2A_ receptor	Protein	Upregulation	Lung tissue in mice with acute lung injury	[15]
P2*X*_3_ receptor	Protein and mRNA	Downregulation	Dorsal root ganglia in rats with type 2 diabetes melitus	[16]
Dopamine D2 receptor	Protein and mRNA	Upregulation	Astrocytes in the middle cerebral artery occlusion (MCAO) mouse model	[17]
mIL-2R	Protein	Inhibit expression	Peripheral blood mononuclear cells	[18]
Histamine	Molecule	Potent release stimulation	Tissue mast cells	[19]
NO	Molecule	Reduce production	Microglial cells	[20]
			Macrophages	[21]
nNOS	Protein	Reduce activity	Cerebral cortex in morphine-dependent, naloxone-precipitated withdrawal rats	[13, 14]
iNOS	mRNA	Downregulation	Microglial cells	[22]
COX-2	mRNA	Downregulation	Microglial cells	[21]
Prostaglandin E2	Protein	Reduce expression	Microglial cells	[22]
			Macrophages	[21]
TNF	Protein and mRNA	Downregulation	Microglial cells	[22]
			RBL-2H3 cells	[23]
			Synoviocytes	[24]
INF-*γ*	Protein	Reduce production	Spleen cells	[25]
			Serum from mesangial proliferative nephritis patients	[18]
IL-6	Protein	Reduce production	Macrophages	[26]
		Enhance production	Peripheral blood mononuclear cells	[27]
IL-1 and IL1-*β*	Protein	Reduce production	Serum of CIA rats/macrophages	[28]
IL-4	Protein	Reduce production	Antigen-activated RBL-2H3 cells	[23]
IL-5	Protein	Reduce production	Spleen cells	[25]
IL-8	Protein	Inhibit production	Peripheral blood mononuclear cells	[27]
IL-18	mRNA	Downregulation	Brain tissue	[17]
IL-13	mRNA	Downregulation	Human synovial sarcoma	[28]
IL-10	Protein	Upregulation	Rat serum	[29]
IL-17A	Protein	Downregulation	Rat serum	[29]
Leukotriene C4	Lipophilic molecule	Reduce production	Macrophages	[21]
NF-*κ*B	Protein	Inhibition activity	Monocyte-derived dendritic cells	[30]
			Macrophage and synoviocytes	[24]
P38 MAPK	Protein	Reduce phosphorylation	Antigen-activated RBL-2H3 cells	[23]
ERK	Protein	Activation	Macrophages	[2]
*β*-Hexosaminidase	Protein	Reduce release	Mast cell mediated by Fc*ε*RI	[23]
HO-1	Protein	Induce expression	Rat liver tissue	[31]
TGF-*β*	Protein	Enhance secretion	Spleen cells	[32]
VCAM-1	Molecule	Reduce production	Fibroblast-like synoviocytes	[33]
CCL2	Protein	Inhibit expression	Fibroblast-like synoviocytes	[33]
CXCL8	Protein	Inhibit expression	Fibroblast-like synoviocytes	[33]
T-bet	mRNA	Downregulation expression	Peripheral blood mononuclear cells	[18]
MMP-2 and MMP-9	Protein and mRNA	Inhibit activity and downregulation	Rat paw tissues	[26]
TIMP-1 and TIMP-3	Protein and mRNA	Enhance activity and upregulation	Rat paw tissues	[26]
ROS	Molecule	Reduce production	Microglial cells	[22]
Nrf2	Protein	Upregulation	Spinal cord in rats with spinal cord injury	[34]
NADPH oxidase	Protein	Inhibition of activity	Microglial cells	[22]
I*κ*B-*α*	mRNA and protein	Downregulation and inhibit phosphorylation	Macrophages and synoviocytes	[24]
P-NMDAR1 and NMDAR1	Protein	Reduce expression	Morphine-treated SH-SY5Y cells	[35]
P-CAMKII and CAMKII	Protein	Reduce expression	Morphine-treated SH-SY5Y cells	[35]
PKB	Protein	Reduce phosphorylation	Antigen-activated RBL-2H3 cells	[23]
Gab2	Protein	Reduce phosphorylation	Antigen-activated RBL-2H3 cells	[23]
P-CREB and CREB	Protein	Reduce expression	Morphine-treated SH-SY5Y cells	[35]

GABA, *γ*-amino butyric acid; cAMP, cyclic adenosine monophosphate; mIL-2R, membrane interleukin-2 receptor; NO, nitric oxide; nNOS, neuronal nitric oxide synthase; iNOS, inducible nitric oxide synthase; COX-2, cyclooxygenase-2; TNF, tumor necrosis factor; INF-*γ*, interferon-*γ*; IL-6, interleukin 6; IL-1, interleukin 1; IL1-*β*, interleukin 1-*β*; IL-4, interleukin 4; IL-5, interleukin 5; IL-8, interleukin 8; IL-18, interleukin 18; IL-13, interleukin 13; IL-10, interleukin 10; IL-17A, interleukin 17A; NF-*κ*B, transcription factor nuclear factor kappa B; P38 MAPK, p38 mitogen-activated protein kinase; ERK, extracellular-regulated protein kinase; HO-1, heme oxygenase-1; TGF-*β*, transforming growth factor-*β*; VCAM-1, vascular cell adhesion molecule-1; CCL2, C-C motif ligand 2; CXCL8, C-X-C motif chemokine ligand 8; T-bet, T-box transcription factor expressed in T-cells; MMP-2, matrix metalloproteinase 2; MMP-9, matrix metalloproteinase 9; TIMP-1, tissue inhibitor of metalloproteinase 1; TIMP-3, tissue inhibitor of metalloproteinase 3; ROS, reactive oxygen species; Nrf2, nuclear factor erythroid-2-related factor 2; NADPH, nicotinamide adenine dinucleotide phosphate; I*κ*B-*α*, nuclear factor of kappa light polypeptide gene enhancer in B-cells inhibitor alpha; P-NMDAR1, phosphor-N-methyl-D-aspartate receptor 1; NMDAR1, N-methyl-D-aspartate receptor 1; P-CAMKII, phosphor-calmodulin-dependent protein kinase II; CAMKII, calmodulin-dependent protein kinase II; PKB, protein kinase B; Gab2, GRB2-associated-binding protein 2; P-CREB, phosphor-cAMP-response element-binding protein; CREB, cAMP-response element-binding protein.
